# 3D Printing of Virucidal Polymer Nanocomposites (PLA/Copper Nanoparticles)

**DOI:** 10.3390/polym17030283

**Published:** 2025-01-22

**Authors:** Waldeir Silva Dias, Luana Cristiny da Cruz Demosthenes, João Carlos Martins da Costa, Leandro Aparecido Pocrifka, Nayra Reis do Nascimento, Samantha Coelho Pinheiro, Gilberto Garcia del Pino, José Luis Valin Rivera, Meylí Valin Fernández, José Costa de Macêdo Neto

**Affiliations:** 1 Programa de Pós-Graduação em Ciência e Engenharia de Materiais—PPGCEM, Universidade Federal do Amazonas (UFAM), Manaus 69067-005, AM, Brazil; 2 Programa de Pós-Graduação em Ciência e Engenharia de Materiais—PPGCEM, Instituto Militar de Engenharia (IME), Rio de Janeiro 22290-270, RJ, Brazil; lccd.eng@ime.eb.br; 3 Grupo de Eletroquímica e Materiais Avançados—GEMATA, Departamento de Química, Universidade Federal do Amazonas (UFAM), Manaus 69067-005, AM, Brazil; jcarlosmartins.89@gmail.com (J.C.M.d.C.); pocrifka@gmail.com (L.A.P.); 4 Instituto Conecthus—Tecnologia e Biotecnologia do Amazonas, Manaus 69075-000, AM, Brazil; dra.nayrareis@gmail.com; 5 Departamento de Engenharia Civil, Universidade do Estado do Amazonas (UEA), Manaus 69050-020, AM, Brazil; spinheiro@uea.edu.br; 6 Departamento de Engenharia Mecânica, Universidade do Estado do Amazonas (UEA), Manaus 69050-020, AM, Brazil; gpino@uea.edu.br; 7 Escuela de Ingeniería Mecánica, Pontificia Universidad Católica de Valparaíso, Concepción 2430000, Chile; jose.valin@pucv.cl; 8 Department of Mechanical Engineering (DIM), Faculty of Engineering (FI), University of Concepción, Concepción 4070409, Chile; mvalin@udec.cl; 9 Departamento de Engenharia de Materiais, Universidade do Estado do Amazonas (UEA), Manaus 69050-020, AM, Brazil; jmacedo@uea.edu.br

**Keywords:** 3D printing, nanoparticles, copper, virucidal, polylactic acid (PLA)

## Abstract

Metallic nanoparticles with virucidal properties dispersed in a polymeric matrix have gained prominence in the scientific community as a rapid and effective alternative that employs the additive manufacturing (AM) or 3D printing method. This study aims to produce filaments for 3D printing using polymer nanocomposites based on polylactic acid (PLA) and copper nanoparticles (CuNPs) in different proportions. The virucidal activity of various proportions of nanoparticles in PLA was investigated. The composites were produced following a mixture design (DOE) with concentrations ranging from 1% to 2% copper nanoparticles, which were blended with PLA using a single-screw extruder. The samples were characterized by thermogravimetry (TG), differential scanning calorimetry (DSC), tensile strength testing, and fracture analysis using scanning electron microscopy (SEM). A thermal analysis of the composites indicated that the CuNPs contributed to an increase in the degradation temperature and crystallization of the PLA. Sample S7 (1.25% of CuNPs) exhibited a 4% increase in the degradation temperature compared to pure PLA. The best tensile strength results were observed in sample S7 (1.25% of CuNPs), 30% more than sample S3 (1.33% of CuNPs) due to good material cohesion, as evidenced by microscopy analyses. Regarding virucidal analyses, most composites demonstrated virus inhibition activity.

## 1. Introduction

The novel coronavirus emerged in the city of Wuhan, China caused a severe respiratory illness defined as Severe Acute Respiratory Syndrome Coronavirus 2 (SARS-CoV-2) or coronavirus-2019 (COVID-19). Due to the significant increase in the number of COVID-19 cases, the World Health Organization declared it a pandemic on 11 March 2020 [1]. COVID-19 can be transmitted through aerosols, physical contact between people, and between contaminated skin and high-contact surfaces, such as electronic boxes, cell phones, ID cards, and other items [2,3,4].

Given the current state of COVID-19 contamination and its new variants, research into materials that resist surface contamination has intensified. To address this issue, surfaces and coatings capable of minimizing the presence of active pathogenic viruses are being researched for application in various configurations, such as face masks, to reduce human exposure and mitigate the spread of infectious diseases [5,6]. An area of great importance in the transmission of infectious diseases is the ability of microbes to survive on surfaces, both in healthcare environments and common settings [7].

Some materials, such as stainless steel, metal, gold, and copper, have virucidal properties [8,9,10,11,12]. Copper is one of the two most widely recognized antibacterial and virucidal materials used and characterized at this time. This material has been used in medicine, as an antiseptic and anti-inflammatory agent, for thousands of years. Widespread use is scientifically justified by modern research that identifies several antimicrobial mechanisms for copper. One mechanism is membrane rupture due to electrostatic forces exerted by copper ions on the external plasma membrane of cells [7].

A method for producing polymeric nanocomposites with virus resistance involves the use of nanofillers, nanoparticles with virucidal properties, such as Ag, TiO2, ZnO, and Cu. These materials can be applied to high-contact surfaces in public and hospital environments, such as doorknobs, tables, chairs, floors, and railings [13].

Many of these high-contact objects, such as chairs, tables, floors, and others, can be produced through traditional processes, like plastic injection molding and extrusion. However, one of the most promising processes in recent years is additive manufacturing or 3D printing. This process is related to a group of techniques that are based on the making of objects in three dimensions, making it possible to make materials and giving the possibility of producing more complex shapes [14]. The main materials used in this category of machines are the thermoplastic filaments of polylactic acid (PLA) and polyethylene terephthalate glycol (PETG) [15].

Several studies have been conducted to evaluate these materials, such as the research by Rocha [16], where copper nanoparticles were applied in antifungal activities and as catalysts in “click-chemistry” reactions. This allowed for the production of the colloidal solutions of copper nanoparticles synthesized in flow, where light dispersion confirmed the Tyndall effect caused by the colloids. The synthesized CuNPs demonstrated potential catalytic action. In a comparative test, using the same reagents, time, and conditions, the reaction with the nanoparticles yielded 40%, while the reaction without the CuNPs yielded 18%.

A literature review aimed at identifying the antiviral activity of polylactic–glycolic acid (PLGA) nanosponges and silver nanoparticles (AgNPs) in the neutralization of SARS-CoV-2 found that silver nanoparticles with a diameter of around 10 nm were capable of inhibiting SARS-CoV-2 at concentrations ranging from 1 to 10 parts per million (ppm). Additionally, a 99% inactivation of SARS-CoV-2 was observed with silver nanoparticles (AgNPs) after 2 min of contact, likely related to the coupling of silver-based nanoparticles with viral surface spike glycoproteins rich in sulfhydryl groups, which cleave disulfide bonds to disrupt the SARS-CoV-2 spike protein interaction with the angiotensin converting enzyme 2 (ACE2) receptor [17].

Studies have also evaluated the use of PLA and high-density polyethylene polymers by incorporating graphene and titanium oxide nanoparticles into these matrices. It was observed that the temperature stability was superior for samples containing nanoparticles. A new mechanical behavior was noted when samples were produced with nanoparticles and coatings. Samples with 90% PLA and 1% nanoparticles exhibited maximum tensile strength, and elongation reached a remarkable level when the samples were coated with graphene. This highlights the potential for achieving satisfactory mechanical properties using nanoparticles for material processing through additive manufacturing [18].

In this context, this study aims to differentiate itself from other studies, investigating and analyzing the thermal, mechanical, and virucidal effects caused by the addition of varying amounts of copper nanoparticles (CuNPs) in a PLA matrix to produce filaments for 3D printing. We have few studies that present these percentage levels of CuNPs and that target a virucidal material with application for 3D printing. This study opens the opportunity to manufacture items with the need for virucidal characteristics and with more complex designs through additive manufacturing.

## 2. Materials and Methods

A filament for 3D printing made of polylactic acid (PLA) (PLA Plus, 3DM da Amazônia, Brazil) with a diameter of 1.75 ± 0.05 mm, density of 1.24 g/cm^3^, flowability 210 °C—2.16 kg (ASTM D1238), point of temperature of 165–180 °C (ASTM D3418), and glass transition temperature of 55–60 °C (ASTM D3418) was used. Copper nanoparticles (CuNPs) (Hwnanomaterial, China) with spherical morphology, a purity of 99.99%, and a diameter of approximately 80–100 nm were used as nanofiller.

### 2.1. Design of Experiments (DOE)

The objective is to study the effect of different concentrations of copper (Cu) nanoparticles (ranging from 1% to 2%) on the properties of the composite material. The independent variable in the study is the concentration of copper nanoparticles, and the dependent variable would be the composite’s performance in terms of these properties. Com a abordagem DOE, você pode determinar a melhor concentração de cobre para otimizar as propriedades do compósito. The experimental design was carried out considering the mixture with extreme vertices with 15 replicates and two mixing factors (PLA and NPCu). The combination between each factor was obtained by Minitab software, version 19.2. Table 1 presents the results of the combination of factors, showing the percentage and weight of the CuNPs and PLA of each mixture that was performed.

### 2.2. Filament Obtaining

By using mechanical blending, it is possible to maintain PLA in its solid state, preserving the desired characteristics for the composite, such as strength, rigidity, and thermal stability. Mechanical blending is an eco-friendlier process, as it does not require the use of hazardous chemicals and generates less toxic waste. Mechanical blending, such as using ball mills or extruders, is generally simpler and less costly compared to the dissolution process followed by solvent removal. Additionally, the operational costs and infrastructure requirements for mechanical blending are lower, especially when compared to procedures involving dissolution, drying, and solvent purification.

**Figure 1:**
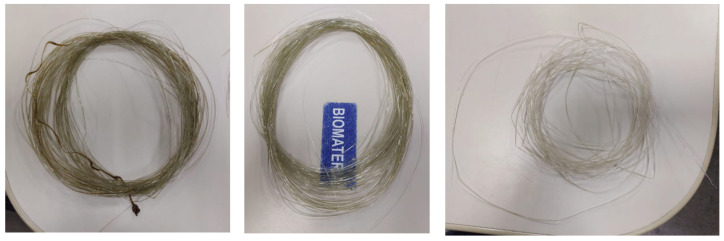
Nanocomposite filaments.

To obtain the nanocomposite filaments (Figure 1), first, the pure PLA filament with a diameter of 1.75 ± 0.05 mm (all filaments performed perfectly on the printer extruders) was crushed in a mini-granulator (AX-Granulador, AX Plástico, Brazil). After crushing, the filament became pellets with an average length of 3.58 ± 0.17 mm. Then, the filament in the form of pellets was heated to 100 °C in an oven for 2 h. After drying, the pellets were manually mixed with the copper nanoparticles in a beaker using a glass rod. A single-screw extruder (AX16LD26MM, Axplástico, Brazil) was used to produce the PLA/NPCu nanocomposite filament. The mixtures were extruded at temperatures of Zone 1: 274 °C, Zone 2: 289 °C, Zone 3: 284 (feed to die), until a filament diameter of 1.75 ± 0.05 mm was obtained. A screw speed of 50 rpm and torque of 26 N·m were used for all mixtures produced, and the filament was cooled at room temperature.

### 2.3. 3D Printing

**Figure 2:**
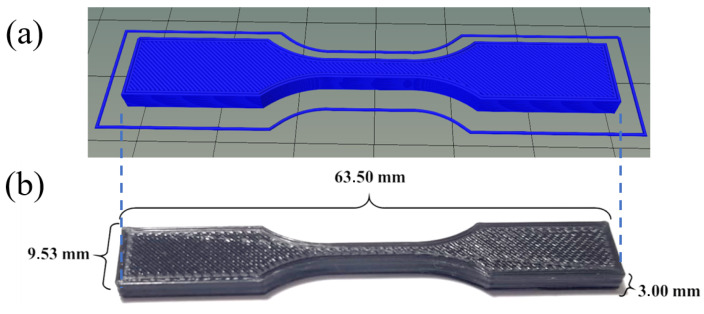
Sample design: (**a**) drawing of the sample to be 3D-printed and (**b**) 3D nanocomposite printed sample.

**Figure 3:**
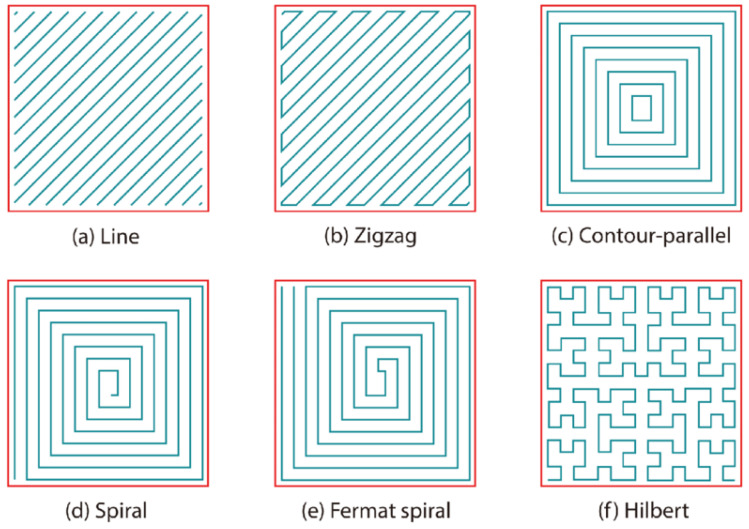
Zigzag pattern 3D printing.

The design of the sample to be obtained through 3D printing and a printed sample are shown in Figure 2. For 3D printing of the specimens, a 3D printer (PCYES FABER 10, PCYES, Brazil) was used, using a nozzle temperature of 220 °C, at a distance of 0.2 mm between the table and the extruder nozzle, 7 min and 41 s of estimated printing time for each specimen, a total of 15 layers of filaments, 5606 of total lines, a filament accuracy of 614 mm, 100% of density, 50 (mm/s) of printing speed, and 45 °C of table temperature. A “zigzag” filling pattern with +45° and −45° deposition was used (Figure 3). The samples were designed using SolidWorks 2021 version and, later, were converted to the STL (standard tesselation language) format. The samples were sliced using Craftware 1.13 beta software, from which the G-code was obtained, before being finalized by Pronterface software. The test specimens were printed with the standards and dimensions of the international standards [19], Type-V using Repetier Host software.

### 2.4. Electron Microscopy

The cohesion and defects in the deposition of filaments on the surfaces and fractures of the samples were evaluated using a scanning electron microscope (SEM) (JEOL IT500HR, Jeol, Japan). The surface of the samples was metallized for 10 min using a device (D II-29010SCTR Smart coater, Jeol, Japan) to perform electron microscopy. The distribution of nanoparticles in the PLA matrix was observed using a transmission electron microscope (TEM) (JEOL 1400, Jeol, Japan). The samples were produced by ultrathin sections (~100 nm) using a microtome (EM UC7, Leica, Germany).

### 2.5. Fourier Transform Infrared Spectroscopy (FTIR)

Fourier transform infrared (FTIR) spectra were obtained using the equipment (IRAffinity-1S, Shimadzu, Japan). The measurements were performed in the range of 4000–400 cm^−1^ and nominal resolution of 4 cm^−1^. The data were obtained using the IRsolution software 1.3 version. The infrared spectrum of the samples was measured by the attenuated total reflection (ATR) method using an accessory (MIRacle-10, Shimadzu, Japan) coupled to the equipment.

### 2.6. DSC and TGA

For this analysis, a device (TG/DTA Seiko, 6200) and the samples in powder form were used. Alumina crucibles were used as sample holders and were heated at 20 °C/min. Heating was performed under an argon atmosphere with a flow rate of 100 mL/min in a range of 100 °C to 900 °C.

### 2.7. Tensile Test

**Figure 4:**
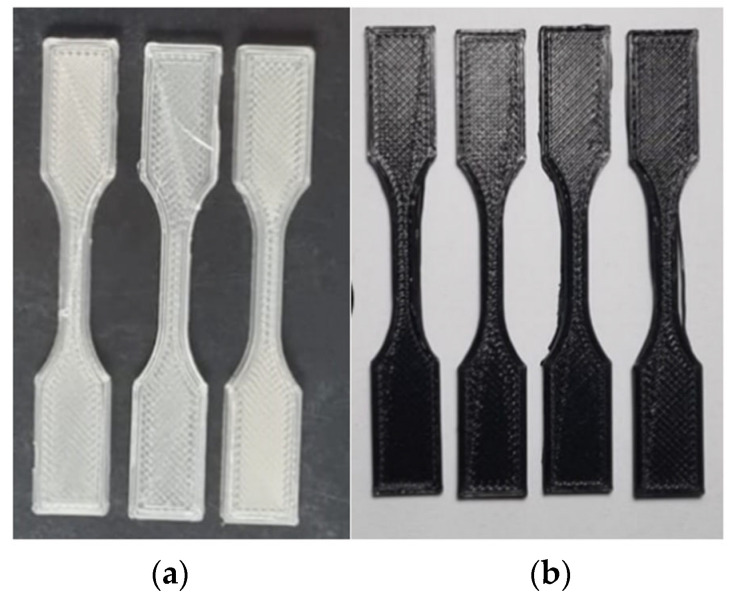
3D-printed samples of the tensile test specimens: (**a**) PLA and (**b**) PLA + CuNP, on the right.

To perform the tensile test, a universal mechanical testing machine (5582, Instron, USA) with a 1.0 kN load cell, a resolution of 0.01 kg.f, and a test speed of 0.5 mm/min was used. The test was performed in a room with a controlled temperature between 23 ± 2 °C, a relative humidity of 50 ± 5%, and a stabilization time of these conditions of more than 3 h [19]. Four specimens were tested for each compositionThe PLA and PLA + CuNP samples can be seen in Figure 4.

### 2.8. Virucidal Activity Test

This analysis aims to evaluate, in accordance with current standards, the inactivation of viral particles in different times after contact with the treated surface/tissue [20]. The following viral models were used: (i) murine hepatitis virus (MHV-3) (enveloped virus model) and (ii) human adenovirus (HAdV-2) (non-enveloped virus model). To perform this test, the virus was placed in direct contact with the samples, which were eluted and applied inside a Petri dish. The dish contained a cell mat with the virus strains. This procedure allowed the infection and replication of the virus in its cavities. The respective strains were (**a**) L929—ATCC CCL-1 CCL-1: rat fibroblast (permissive to MHV-3 and the lineage) and (**b**) A549—ATCC-CCL-185: human lung carcinoma (permissive to HAdV-2). Replicates and duplicates were performed in this assay.

Although there are already studies applying copper nanoparticles in composite materials, few articles and research focus on their application in the 3D printing field, which involves numerous details and various characterization techniques.

## 3. Results

### 3.1. Fourier Transform Infrared Spectroscopy (FTIR)

The bands observed for PLA in the range of 2290 cm^−1^ and PLA/nanoCu S3 at 3011 cm^−1^ correspond to the stretching of C–CH_3_, while the vibration bands of the stretching –CH appeared at 2240 cm^−1^ for PLA and at 2263 cm^−1^ for the nanocomposite. The bands observed in the IVTR spectra at 1747 cm^−1^ and 1765 cm^−1^ are attributed to the stretching modes C=O, while the bands at 1453 and 1469 cm^−1^ are attributed to the bending of CH_3_, and in the medium intensity bands 1358 cm^−1^ (PLA) and 1377 cm^−1^ (PLA/CuNP S3), the deformation is of C–O.

The C=O stretching mode was observed in bands 1181 and 1199 cm^−1^ for PLA and the nanocomposite, in this order. The O–C–O stretching vibration, characteristic of PLA, appears in the band 1128 cm^−1^, while in PLA/CuNP S3, this band occurred at 1144 cm^−1^. Intensity bands at 1082 and 1043 cm^−1^ for PLA and at 1099 and 1059 cm^−1^ for the nanocomposite are attributed to the stretching modes of the C=O carbonyl group. For CH_3_ agitation, the vibration bands were 954 cm^−1^ (PLA) and 970 cm^−1^ (nanocomposite), while the stretching of the methyl group at 868 and 755 cm^−1^ for the polymer and 886 and 756 cm^−1^ for the PLA/CuNP S3 material.

**Figure 5:**
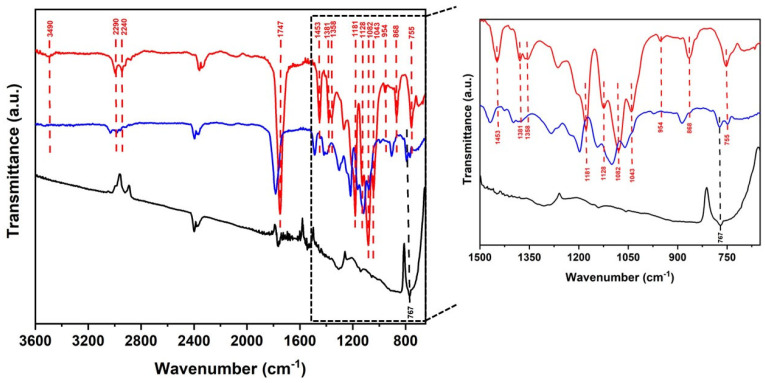
Spectra of the 3D-printed PLA materials and the PLA/CuNP S3 nanocomposite.

The FTIR spectra of the nanocomposite showed characteristics similar to those of pure PLA, and in the band positions, displacements of the connections of the polymer groups were observed (Figure 5). However, the intensities of the bands are reduced in the polymers that were reinforced with copper nanoparticles. This can be explained based on the interactions between Cu nanoparticles and PLA by polymer adsorption on the surfaces of the CH3 methyl group.

The spectrum observed in the nanocomposite at 767 cm^−1^ is related to the stretching of the CuNP bond [21]. Observing the IVTF results, it is confirmed that the copper nanoparticles are incorporated into the PLA matrix, this interaction occurs via adsorption between the matrix and the nanofiller. This contact occurred physically between the polymer and CuNPs, the actions occurred between the surface methyl groups of PLA and the nanoparticles with the methyl group CH_3_, we emphasized that all samples were analyzed, but for comparison purposes, we adopted only sample S3 as a comparative effect. All the behaviors of the bands observed in the study between the matrix and nanofiller have also been reported in the works [22,23,24].

Figure 4 shows the spectra of the PLA 3D printing materials and the PLA/CuNP S3 nanocomposite. All frequencies observed in the materials with their relative intensities of the characteristic bands corresponding to the PLA and nanocomposite materials are summarized in Table 2.

### 3.2. Thermogravimetric Analysis (TGA)

**Figure 6:**
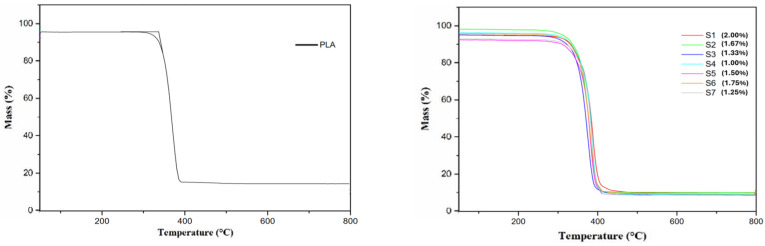
Thermogravimetric analysis of pure PLA sample and PLA samples with addition of copper nanoparticles. Measure the change in mass of a sample as it is heated.

Regarding the thermal properties of PLA and PLA/CuNP composites, thermogravimetric characterization was used to determine the mass loss in relation to temperature, at an increasing temperature rate, to obtain the degradation temperature of the materials. Figure 5 shows the TGA curves for the pure PLA filament. Figure 6 illustrates the PLA composite with the copper fractions. Through these curves, it is possible to determine the mass loss as well as the degradation temperature of the materials under analysis. The mass loss before 200 °C is attributed to the desorption of water molecules, as well as the complete degradation of small impurities present in the material, representing a mass loss of less than 1% of the total mass. The mass loss between 200 °C and 400 °C is associated with the degradation of PLA. The main stage of the thermal decomposition of PLA begins at a temperature of approximately 320 °C and ends at 380 °C, with a peak centered at 362.50 °C and a reduction of 96.4% of the initial mass of the material [27]. Comparing the results obtained with studies in the literature associated with PLA, not limited to additive manufacturing, it was observed that the initial decomposition temperature is very close to the values found in other studies [28], in this case, 342.2 °C, 337.5 °C, and 330 °C, respectively. In other studies, reductions of 95% in the initial mass of PLA were identified at 377 °C [29] and of 98% in the initial mass of PLA at 395 °C [26], results similar to those reported in this work. Higher crystallinity can increase heat resistance, as the ordered structure makes the material more stable to thermal deformation and melting.

The characterization by TGA allows us to draw a coherent conclusion about the percentage by weight of copper distributed over the PLA polymer matrix. Table 3 shows the thermal behavior, by TGA, of the PLA/CuNP composites with different proportions of CuNPs inserted in the matrix. The analysis of Figure 4 showed that the composites degraded at temperatures higher than that of PLA. According to Table 2, considering the degradation temperatures (onset and endset), it is possible to note an increase in these temperatures, and this increase is directly proportional to the percentage of copper inserted in the PLA matrix. Studies indicate that this increase occurs when the nanoparticles are inserted into the PLA chains; an additional restriction will be produced to restrict the mobility of the chain branches [30]. The influence of the temperature will be transmitted from the PLA chains to the CuNPs.

Regarding composites with CuNPs, it is possible to observe three very specific regions. The first of these are found in the region before 200 °C, a second region between 200 °C and 400 °C, and the third region between 400 °C and 800 °C.

It is possible to see that, at the end of Region 2 (Figure 6) (temperature of 380 °C), only copper is present in the characterization. The mass percentages at this temperature in relation to the composites (PLA+CuNP) correspond to the mass percentage of copper present in the composite filament. The final mass loss in thermography, between 380 °C and 800 °C (Region 3—Figure 4), is associated with the oxidation processes of metallic copper by oxygen. The oxidation of copper particles results, for the most part, from the formation of various oxides within the process stages [31]. The most common copper oxides are Cu_2_O and CuO, with Cu_2_O becoming more abundant as the temperature increases above approximately 800 °C, due to its thermal stability.

### 3.3. Differential Scanning Calorimetry (DSC)

**Figure 7:**
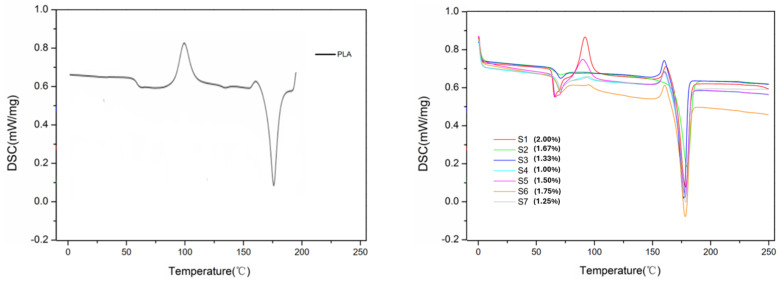
Calorimetric analysis of the pure PLA sample and samples with copper nanoparticles. Measurement of the heat change associated with the thermal denaturation of the molecule when heated at a constant rate.

Another thermal characterization performed was differential scanning calorimetry (DSC). Figure 7 shows the thermal curves of heat flow as a function of time and temperature for PLA and composites with CuNPs. The glass transition temperatures (Tg), initial cold crystallization (Tcc), and initial melting temperature are also shown in Table 4. Figure 7 shows the crystallization from the melt (cooling curve) and the curve of the second heating or reheating of PLA as a function of the temperature. Three events are observed as a function of time, with two being heating and one being cooling. The first event is related to melting during the first heating of the material. After melting the PLA, it cools down, the second event recorded concerns the crystallization of the material from the melt. Subsequently, a second heating occurs, and thus, a new event is shown in the graph, which is cold crystallization, followed by melting the material.

It is shown in Figure 7 and Table 4 that the addition of CuNPs in the PLA matrix results in a notable change towards the glass transition, the Tg of PLA is around 62.7 °C, very close to the Tg found in another study [28]. The PLA and CuNP composites obtained a slight increase in Tg compared to PLA, being between 65 and 70 °C. This indicates that the addition of CuNPs reduced the flexibility of the composite chain in relation to PLA, since Tg is mainly related to the flexibility of the polymer chain. Similar observations were observed in another study [32], which indicated that this process may be related to the hydrogen bonding interaction belonging to the OH group of PLA and CuNPs, something that may induce restriction in the mobility of the polymer chain.

After the occurrence of the Tg temperature, the polymer chains begin to gain mobility and give rise to the first structural atomic rearrangement, characterized by the exothermic first-order transition corresponding to the initial cold crystallization temperature. Based on the data presented in Table 4, the cold crystallization temperature (Tcc) decreased for all composites in relation to PLA, which is at 99.1 °C. This reduction in the Tcc temperature is an indication that crystallization occurs rapidly from the melt.

CuNPs, therefore, demonstrate the same effect as a nucleating agent in the PLA polymer matrix [32]. The increase in Tcc was expected, since the nucleating effect of nanofillers is normally evidenced with the reduction in Tcc [33].

For all samples of the composite with CuNPs, at all heating rates adopted, the phenomenon of cold crystallization occurs. This phenomenon is quite common in PLA and occurs due to its relatively low crystallization rate, due to the rapid cooling of the molten state, with not enough time for crystallization to occur completely [34]. However, as it is a crystallizable polymer that has a rigid chemical structure, during heating, the mobility of the chain increases, and crystallization occurs.

It can be concluded that in the presence of CuNPs, the crystallinity of the composites increases, thus, it can be stated that they favor the crystallization of PLA [35].

The melting temperatures (Tm) between PLA and CuNP composites were different, being around 169–179 °C for all samples (Table 3). In comparison to PLA, there was an increase in Tm. The emergence of the double melting peak was due to the formation of small and imperfect crystals during cooling, which modify through melting and recrystallization at low heating rates [36].

**Figure 8:**
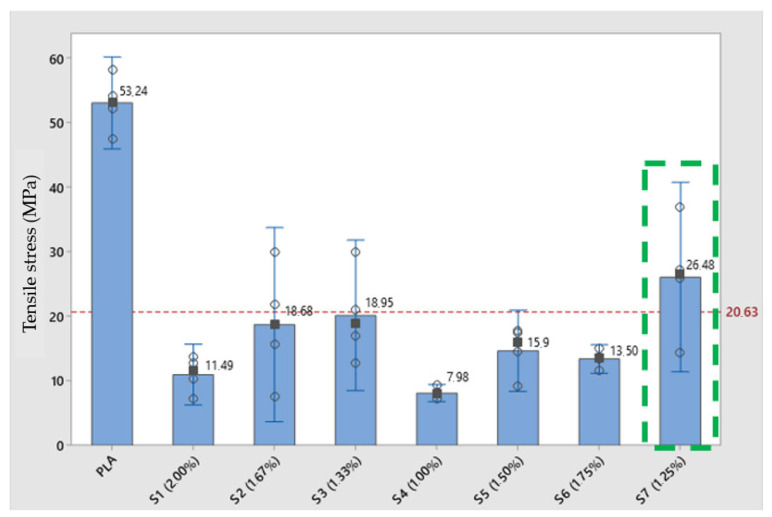
Tensile stress applied to the samples, where it is possible to make a progression of the force per unit area exerted on it, resulting in deformations or changes in its physical properties.

Region 3, obtained in Figure 8, is characterized by the thermal event associated with the first-order endothermic transition corresponding to the initial melting temperature of PLA (Tm) at 169.90 °C, above the Tm found in the study by [37] of 155 °C for the PLA filament.

The effect of processing associated with the CuNP content played an important role in the composite, since the processing flow directly influences the molecular orientation of the material, which influences crystallization, and the resulting structure tends to be stabilized by the interaction between crystallization and relaxation [38].

The increase in the amount of heat required for chemical and/or physical transformations to occur is influenced by the presence of CuNPs in the composite. In general, larger quantities require more heat or energy for a transformation to occur, since the time at a given temperature is reduced. The presence of the nanoparticle tends to reduce mobility, requiring more heat for the transformations to occur. Increased crystallinity indicates improved structural integrity, which means an improvement in the thermal stability of the material, thus opening up an even greater possibility of applying the material to items that require high temperatures in their applications.

### 3.4. Mechanical Properties

The mechanical tensile strength results for pure PLA and nanocomposites are shown in Figure 8. Pure PLA presented a tensile strength of 53.24 MPa, and the nanocomposites presented a significant reduction in relation to PLA. In relation to sample S7 (1.25% copper nanoparticles), which obtained the best result compared to the other composites, we have a reduction in the maximum load of 39% compared to PLA. Sample S3 obtained the second-best result among the composites, presenting a difference of 55% in relation to PLA.

The worsening of the strength properties may result from the partial degradation of PLA. Another possibility for the reduction in the maximum rupture load may be related to the production process of the test specimens, since, as evidenced in the thermal analyses, we had an increase in the melting temperatures (Tg), which can directly influence the quality of the material deposited in the 3D printing process.

The tensile strength values are presented in Table 5 and Table 6 and Figure 8. It can be observed that the nanocomposites presented a reduction in tensile strength in relation to PLA. Sample S7 (CuNP-1.25%) obtained the best result when compared to the other composites and presented above the average value (20.63 MPa) for all samples in the study. However, in relation to pure PLA, there was a 51% reduction in tensile stress. When compared to nanocomposite S3 (CuNP—1.33%), sample S7 presented a tensile strength value 23% higher. In relation to sample S4 (CuNP—1.00%), sample S7 presented a tensile strength 69% higher. It is known from the specialized literature that the greater the amount of PLA%, the greater the tensile strength and Young’s modulus [33,34]. It can be observed that the tensile strength of the samples is directly proportional to the ratio between the two components. PLA has a rigid but brittle polymer chain. Its strength decreases with the introduction of additives dispersed in its polymer matrix. However, it is essential not to exceed certain additive concentrations. It has been observed that the addition of 5–10 wt.% copper to PLA leads to a significant decrease in tensile strength due to the tendency of the particles to agglomerate in the PLA melt [35].

**Figure 9:**
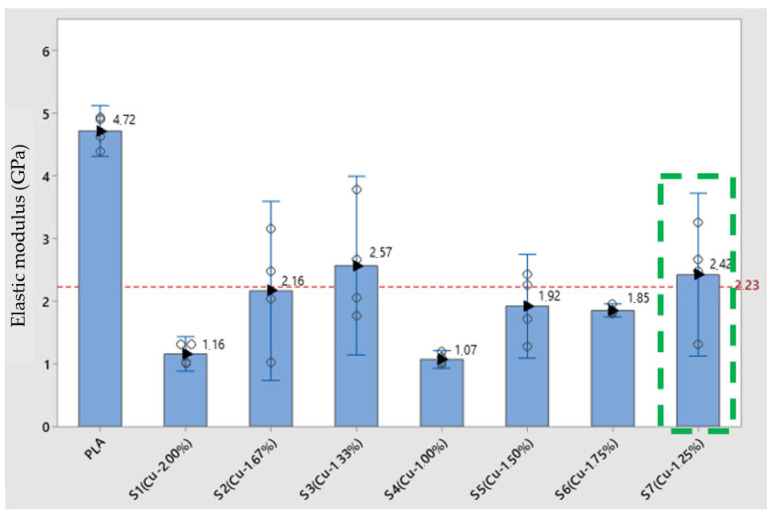
Elastic modulus of the studied samples, pure PLA, and PLA with copper nanoparticles. The elasticity modules are presented and where it is possible to observe the progression of stiffness.

The modulus of elasticity is directly linked to the rigidity of the material, and by analyzing the values of the modulus of elasticity presented in Table 1 and Figure 9, it is noticeable that there was a large reduction in the modulus when comparing the composites and PLA. In relation to the average value of the study of 2.23 GPa, PLA is 53% above this value. In relation to the composites, one sample that stand out the most are sample S3 (CuNP—1.33%), which is 13% above the average value of the analysis of the study samples but is 46% lower than the reference sample, PLA without the addition of CuNPs. Another sample that stands out is S7 (CuNP—1.25%), whose modulus of elasticity value was 8% above the average of the study but 49% below the PLA sample without the addition of CuNPs. This result reflects the reduction in rigidity in the samples with the addition of CuNPs, and this characteristic is directly proportional to the increase in proportions in the composites.

Based on thermal analysis, it is possible to observe changes in transition temperatures in composites, according to the fraction of CuNPs in the composites. It is well-known that the mechanical and physical properties of semicrystalline polymeric materials, such as PLA, are guided by the morphology of the molecular superstructure, which in turn, is controlled by the crystallization process. Thus, the properties of semicrystalline polymers depend on the crystallization behavior, which was evidently altered by the presence of CuNPs. The addition of nanofillers to the matrix influences the crystallization behavior and, consequently, modifies the mechanical and thermal properties compared to pure PLA. In the field of nanoscience, nanocomposites interfere with the mechanical properties and heat distortion temperature. All this influence of the crystallization process acts directly on the characteristics of additive manufacturing (3D printing) of composites [33,34].

From the tensile test investigation, we can deduce that the flexibility of the samples decreases as the amount of PLA decreases. The modulus of elasticity of the samples is directly proportional to the maximum force supported and inversely proportional to the elongation. Thus, the sample with a greater amount of PLA presents greater resistance and force supported, being more rigid than the samples with higher levels of copper nanoparticles. As the concentrations of CuNPs increase, there is a higher likelihood of nanoparticle agglomeration, forming clusters. These agglomerates can act as weak points in the material, as the interface between the aggregated particles and the polymer matrix may not be well-adhered. As a result, the load transfer between PLA and cooper nanoparticles is impaired due to this agglomeration of particles, which causes the tensile strength of the composite to decrease.

### 3.5. Fracture of Samples Obtained by 3D Printing

To demonstrate the behavior of the samples in relation to the proportions of copper present in the PLA matrix, and how the processing of the material influenced its properties, a systematic analysis of its morphologies was performed.

**Figure 10:**
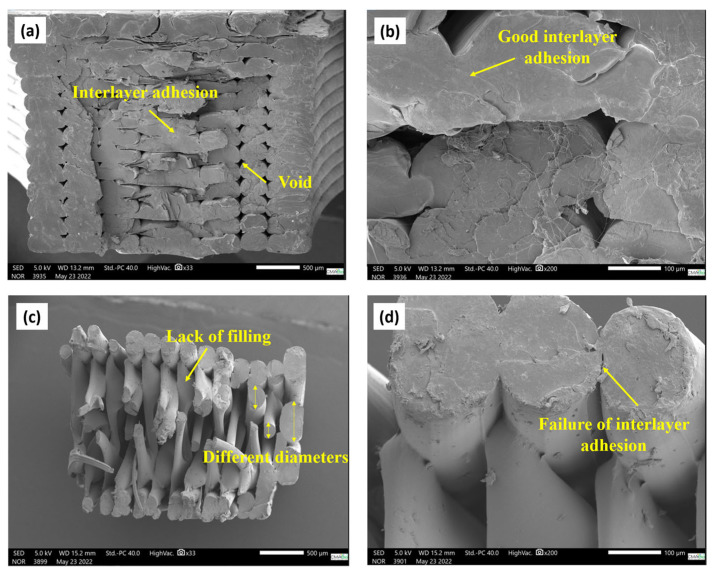

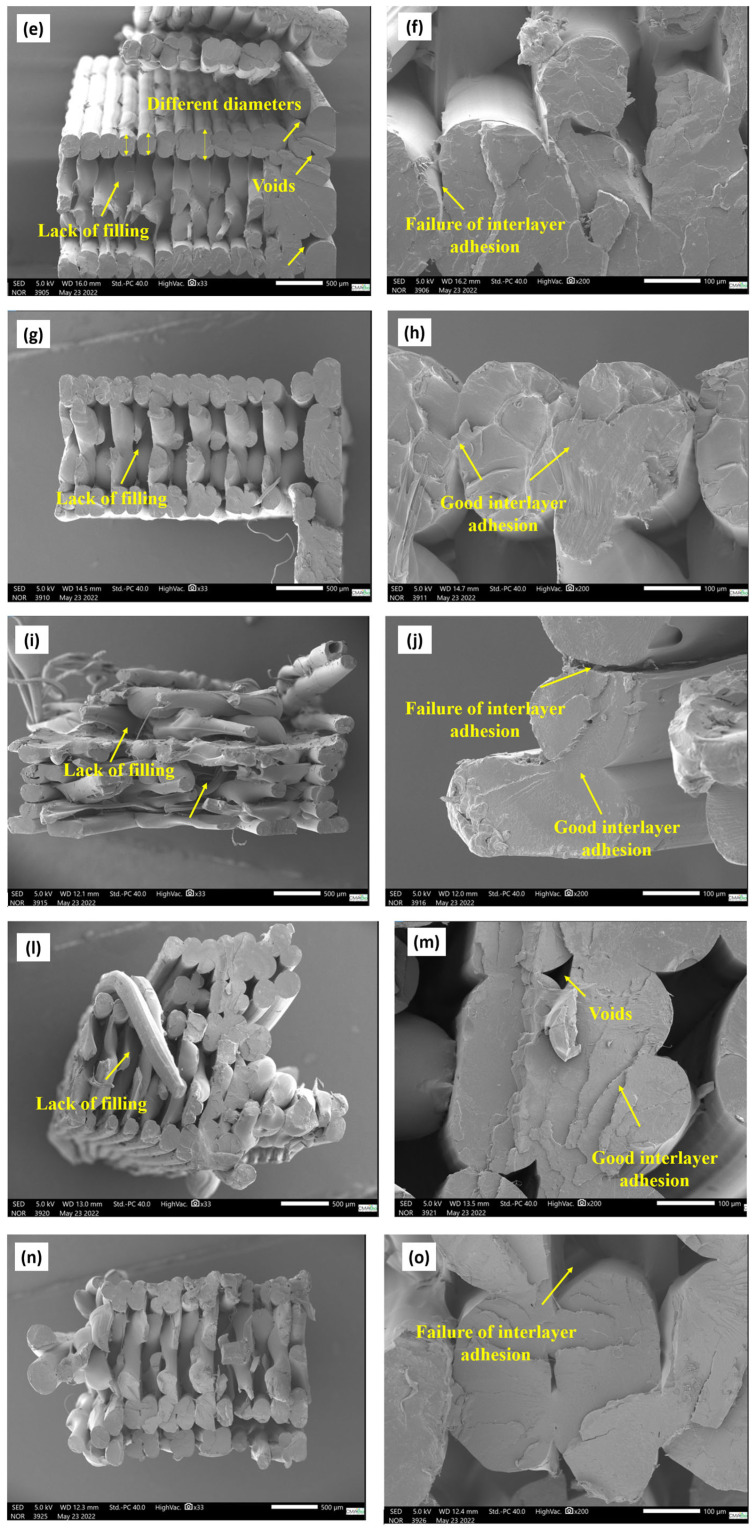

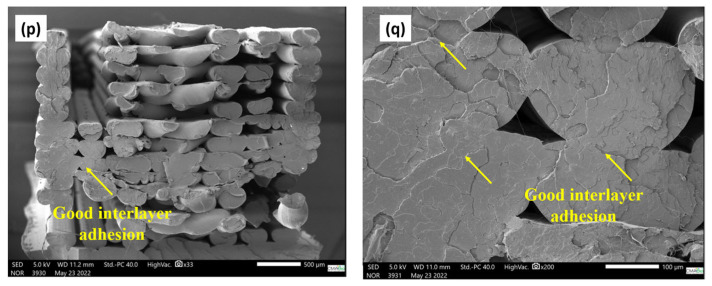
SEM micrographs of the fracture surface of PLA tensile strength specimens (**a**) 33× and (**b**) 200×/S1 (CuNPs—2%) (**c**) 33× and (**d**) 200×/S2 (CuNPs—1.67%) (**e**) 33× and (**f**) 200×/S3 (CuNPs—1.33%) (**g**) 33× and (**h**) 200×/S4 (nanoCu—1.00%) (**i**) 33× and (**j**) 200×/S5 (CuNP—1.50%) (**l**) 33× and (**m**) 200×/S6 (CuNPs—1.75%) (**n**) 33× and (**o**) 200×/S7 (CuNPs—1.25%) (**p**) 33× and (**q**) 200×.

Figure 10 shows the micrographs of the fracture surface of the PLA without the addition of CuNPs. It is notable that the sample printed in pure PLA “neat” obtained better cohesion between the deposited filaments than the nanocomposite filaments. Areas with an excellent connection between the filaments, thus, contributed to the distribution of the tensile stresses in a larger filling area, and this fact directly contributed to the performance of the samples in the mechanical analyses: modulus of elasticity 4.72 GPA, tensile stress 53.06 MPa, maximum tensile load of 472.41 N.

The quality of the union between adjacent filaments is an important factor in determining the mechanical properties of parts obtained by AM [39,40]. The analysis of the results found in the research, for the mechanical properties of traction and hardness of the material, associated with the SEM images, allows us to prove that the reductions in these mechanical properties were determined according to the insertion of CuNPs. Considering the results of the masses, samples S4 (CuNPs—1.00%), S1 (CuNPs—2.00%), S6 (CuNPs—1.75%), and S5 (CuNPs—1.50%) (Figure 9) presented the lowest values of tensile stress and modulus of elasticity and the greatest dispersion of values considering the average in the tests. A comparison between the maximum load at rupture found in samples S4, S1, S6, and S5 shows a reduction in the value found in comparison to PLA of 84%, 76%, 70.4%, and 70.1%, respectively. This characteristic is mainly due to the lack of connections between the filaments deposited in these samples and the failures in the filling, as illustrated in Figure 10. The composites where the highest average values of the rupture stress were obtained, S3 (CuNP—1.33%) and S7 (CuNP—1.25%), presented a better quality in the connection between the neighboring filaments Figure 9 when compared to sample S1; for example, the maximum load at rupture for sample S3 was 25% higher than sample S1. The sample S7 in relation to the maximum load at rupture was 59% higher than sample S1. To illustrate the influence of the quality of the connection between the filaments on the mechanical resistance, in sample S1, the CPs obtained the lowest breaking stress value when compared to the other values, presenting failures in the connection between the filaments and voids in the structure (Figure 10).

Based on the preliminary mechanical and morphological results obtained, it is possible to note the lack of efficiency in the cohesion of the material, according to the increase in CuNPs in the polymer matrix. The optimum contents for the composite are 1.33% by mass of CuNPs (S3) and 1.25% by mass of CuNPs (S7).

### 3.6. Virucidal Activity Test Analysis

After the appropriate infection time, if the viruses are inactivated by the sample under analysis, the cells do not undergo the replication and infection process. If the viruses have been replicated successfully, the cells will present replication and an evident cytopathic effect, which is the lysis (rupture) of the cell. Therefore, based on Table 7 the viruses are diluted in wells so that their numerical quantity decreases on a logarithmic scale. Therefore, there are wells with 10^2^ viral particles (100), 10³ viral particles (1000), 10^4^ viral particles (10,000), and 10^5^ viral particles (100,000). The inhibition of virus activity occurred, and the cell carpet appeared intact and healthy; therefore, the inactivation of 1 log of viruses was observed, thus indicating the percentage of inactivated viruses/viral inhibition in 3 h. For inhibition to occur, the inhibited logs are counted; therefore, 3 logs represented 99.9% of the total virus present (in the experiment performed, there were 5 logs). The results regarding the virucidal response of the material are presented in Table 5.

**Figure 11:**
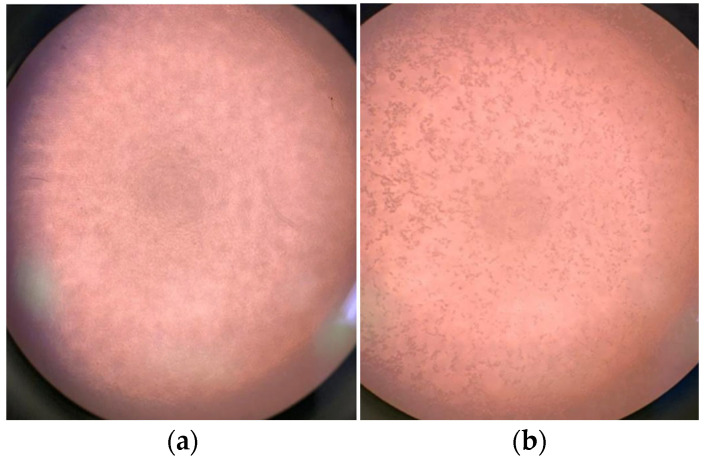
Effectiveness of CuNPs in samples: (**a**) monolayer of cell line without cytopathic effect and (**b**) monolayer after undergoing cytopathic effect.

Regarding the results, only sample S4, which contains the lowest concentration of CuNPs (1.00%) among all samples in the study, was not found to inhibit viral multiplication within host cells, preventing replication or blocking the entry of viral particles into host cells. The mechanism behind the antiviral effect of CuNPs interferes with viral replication by two separate mechanisms. CuNPs can bind to the sulfur-containing residues on the surface glycoproteins of the virus, thereby blocking the host cell from binding and penetrating the virus, thus leaving the virus in the extracellular space where it is unable to propagate [41]. CuNPs can cross the cell membrane and interact with viral factors and double-stranded DNA, thus blocking viral replication and the proper assembly of viral progeny. CuNPs showed its efficacy in the samples produced; Figure 11 shows the difference between samples with a cytopathic effect and another without the effect.

### 3.7. Electron Microscopy

**Figure 12:**
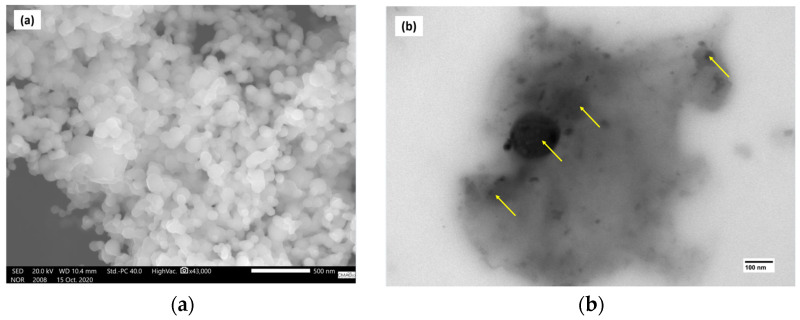
Image obtained by electron microscopy: (**a**) copper nanoparticles and (**b**) polymeric nanocomposite (S7).

Figure 12a shows the image obtained by SEM. The image shows the copper nanoparticles with spherical morphology and are arranged in an agglomerated manner with dimensions between 80 and 100 nm, which agrees with the literature. Figure 12b shows the image obtained by the TEM of the nanocomposite (S7) that presented the best mechanical properties. The image shows the copper nanoparticles well distributed in the PLA matrix, and this resulted in an improvement in the mechanical properties.

## 4. Conclusions

This study conducted a study of the performance of polymeric material with the insertion of CuNPs with the aim of increasing the mechanical performance and virucidal properties for the manufacture of parts by additive manufacturing, using the material extrusion principle. In this context, several process parameters, the characteristics of PLA nanocomposites, and the construction of parts were presented.

Regarding virucidal activity, it was possible to observe that, above the composition of 1% CuNPs, we already have good responses to the antiviral process. The mechanism by which CuNPs exert their virucidal action on the MHV-3 and HAdV-2 viruses appears to be related to the binding and damage of the viral envelope of these compositions above a 1% CuNP mass concentration.

Among some points to be evaluated in future work, we can highlight the deposition of the material, which is related to the temperature of the extruder nozzle. This was also observed in the analysis of the SEM images, which shows low interfacial adhesion between the deposited filaments of the nanocomposites.

Regarding the thermal analysis, it was observed that the extrusion temperature (200 °C) was insufficient for processing the composites, according to the presence of CuNPs. When compared to PLA, the melting temperature of the material (Tg) had its degree increased proportionally to the insertion of CuNPs. Considering the TGA analysis, the greatest loss of mass of the composite materials is concentrated between 345 and 404 °C. The degradation of PLA begins around 320 °C. According to the DSC, the degradation of the composite filament begins at a higher temperature compared to the virgin PLA filament. Therefore, the thermal analysis of the composite filament is considered sufficient for MA printing. The DSC also revealed important information regarding the degree of crystallinity of both materials. The addition of CuNPs caused an increase in the degree of crystallinity, indicating that the micronized particles of this material acted as nucleating agents for crystallization.

The morphological analysis showed that the PLA mixture modifies the PLA structure. A study is also suggested regarding the addition of plasticizers or compatibilizing agents in order to improve the bond between the PLA interface and the copper nanoparticles.

Regarding the mechanical analyses, the composites in general did not present good results, with the exceptions being samples S3 and S7. We had greater fragility of the samples, largely due to the influence of the melting temperature of the material in relation to the processing, which resulted in a low filling rate of the test specimen.

Based on the analyses, it was possible to observe that, above the CuNP composition of 1%, we already have good responses to the virucidal action. Regarding the thermal analysis, it was possible to observe the influences of the extrusion and additive manufacturing processes, since the melting temperature of the material (Tg) increased proportionally to the insertion of CuNPs. The addition of CuNPs caused an increase in the degree of crystallinity, indicating that the nanometric particles of this material acted as nucleation agents for crystallization. It is possible to observe such activity through thermal analyses using TG and DSC. The mechanical and thermal properties of the nanocomposites were inferior to those of pure PLA.

## 5. Future Works

A possible improvement would be to investigate more efficient dispersion methods of copper nanoparticles (CuNP) in the PLA matrix, such as the use of ultrasound or high-energy processing. Additionally, it would be interesting to explore the use of compatibilizing agents or plasticizers that could improve the adhesion between CuNPs and PLA. This could result in better interaction between the composite phases, enhance mechanical properties, and reduce the brittleness observed in the samples. A more in-depth study of the influence of different CuNP concentrations on the mechanical and thermal properties of the composites would be useful. The research could evaluate how variations in CuNP concentration affect tensile strength, toughness, deformation, and thermal properties, such as thermal stability and crystallinity. This study could also include an analysis of how these properties evolve over time and with use, considering aging or exposure to different environmental conditions. Given the virucidal activity observed with the addition of CuNPs, the next step would be to investigate the introduction of other materials or additives with antimicrobial properties, such as metal oxides or other nanotubes, to enhance the virucidal action. The development of multicomponent composite materials with improved antiviral properties could be useful for applications in medical devices, protective systems, and other fields related to public health. Further testing with other types of viruses could also expand the understanding of the material’s virucidal effectiveness.

## Figures and Tables

**Table 1:** Result of the experimental planning CuNP x PLA.

Specimen	Cu NPCu (%)	PLA (%)	CuNP (g)	PLA (g)
S1	2.00	98.00	0.40	19.60
S2	1.67	98.33	0.33	19.67
S3	1.33	98.67	0.27	19.73
S4	1.00	99.00	0.20	19.80
S5	1.50	98.50	0.30	19.70
S6	1.75	98.25	0.35	19.65
S7	1.25	98.75	0.25	19.75

**Table 2:** Identification of bands related to PLA and PLA/CuNP S3 nanocomposites.

Wavenumber (cm^−1^)		Task/Assignment
PLA	PLA/CuNP	
2290	3011	Stretching C-CH_3_ [22,23,25,26]
2240	2263	Stretching -CH [22,23,26]
1747	1765	Stretching C=O [22,23,25,26]
1453	1469	Bending CH_3_ [22,23,26]
1381	1400	Bending CH_3_ [22,23,26]
1358	1377	Deformation C-O [22,23,25,26]
1181	1199	Strain C=O [22,23,25,26]
1128	1144	Strain C-O-C [22,23,25,26]
1082	1099	Stretching C=O [22,23,25,26]
1043	1059	Stretching C=O [22,23,25,26]
954	970	Agitation CH_3_ [26]
868	886	Stretching CH_3_ [26]
-	767	Stretching Cu [21]
755	756	Stretching CH_3_ [26]

**Table 3:** Thermal behavior of samples.

Specimens	Thermal Decomposition Temperature (°C)			
	T_onset_	T_endset_	Td	Loss (800 °C)
PLA	320.15	376.80	362.50	96.40%
S1 (2.00)	369.28	404.78	385.64	98.18%
S2 (1.67%)	351.29	399.33	375.01	98.23%
S3 (1.33%)	345.79	391.25	366.20	99.80%
S4 (1.00%)	337.13	387.53	381.69	99.13%
S5 (1.50%)	351.82	397.39	373.40	98.78%
S6 (1.75%)	352.79	398.66	376.10	98.19%
S7 (1.25%)	345.16	391.87	368.99	98.65%

**Table 4:** Glass transition temperatures (Tg), initial cold crystallization temperature (Tcc), and initial melting temperature of PLA and PLA samples with copper nanoparticles.

Specimen	Tg (°C)	Tcc (°C)	Tm (°C)
PLA	62.70	99.10	169.90
S1 (2.00)	65.68	95.08	179.87
S2 (1.67%)	70.76	93.33	178.22
S3 (1.33%)	70.59	88.53	177.64
S4 (1.00%)	70.54	87.33	176.73
S5 (1.50%)	66.37	89.96	177.76
S6 (1.75%)	70.71	94.71	178.45
S7 (1.25%)	70.56	88.71	177.01

**Table 5:** Tensile stress of the specimens.

	Tensile Strength (MPa)		
Specimen	Average	Median	Standard Deviation
PLA	53.06	53.25	4.48
S1 (2.00%)	10.96	11.49	2.96
S2 (1.67%)	18.71	18.69	9.45
S3 (1.33%)	20.15	18.95	7.34
S4 (1.00%)	8.09	7.99	0.84
S5 (1.50%)	14.65	15.90	3.96
S6 (1.75%)	13.39	13.51	1.39
S7 (1.25%)	26.07	26.48	9.23

**Table 6:** Elastic modulus of the studied specimens, pure PLA, and PLA with copper nanoparticles.

	Elastic Modulus (GPa)		
Specimen	Average	Median	Standard Deviation
PLA	4.72	4.77	0.26
S1 (2.00%)	1.16	1.17	0.17
S2 (1.67%)	2.17	2.25	0.90
S3 (1.33%)	2.57	2.36	0.90
S4 (1.00%)	1.07	1.06	0.09
S5 (1.50%)	1.92	1.99	0.52
S6 (1.75%)	1.86	1.84	0.07
S7 (1.25%)	2.43	2.57	0.82

**Table 7:** Results of viral inactivation of the samples.

Specimen	MHV-3	HAdV-2
PLA	NR	NR
S1 (2.00%)	99.9% reduction	99.9% reduction
S2 (1.67%)	99.9% reduction	99.9% reduction
S3 (1.33%)	99.9% reduction	99.9% reduction
S4 (1.00%)	No virus inhibition	No virus inhibition
S5 (1.50%)	99.9% reduction	99.9% reduction
S6 (1.75%)	99.9% reduction	99.9% reduction
S7 (1.25%)	99.9% reduction	99.9% reduction

Legend: NR: not reacted.

## Data Availability

The original contributions presented in this study are included in the article. Further inquiries can be directed to the corresponding authors.
